# A Directory

**Published:** 1881-10

**Authors:** 


### A DIRECTORY.

A Directory of the Dentists practicing in the State of Georgia, July, 1881. Complied by Dr. George H. Whitaker, Sandersville, Ga.

By this directory it appears that there are two hundred and thirty dentists in regular practice in the State of Georgia. This is a work which the profession in every State in the Union should do. It seems to be a necessity, for every State that has a law to regulate the practice of dentistry. There are many advantages that would accrue from having a complete list of the dentists of the country. The American Dental Association has made a very commendable move in this direction; this work of Dr. Whitaker is in answer to that call. It is to be hoped that every State will respond as promptly. The editor will be glad to receive a copy of every such list that may be prepared.

